# 7α,15α-Dibromo-8,16-diphenyl-6,7,14,15-tetra­hydro-6α,14α-epithio­cyclo­octa­[1,2-*b*:5,6-*b*′]diquinoline deuterochloro­form solvate

**DOI:** 10.1107/S1600536809032978

**Published:** 2009-08-26

**Authors:** Isa Y. H. Chan, Roger Bishop, Donald C. Craig, Mohan M. Bhadbhade, Marcia L. Scudder

**Affiliations:** a School of Chemistry, University of New South Wales, Sydney 2052, Australia; b The Analytical Centre, University of New South Wales, Sydney 2052, Australia

## Abstract

In the racemic title compound, C_34_H_22_Br_2_N_2_S·CDCl_3_, pairs of diquinoline host mol­ecules form centrosymmetric brick-like dimers utilizing three different aryl edge-to-face inter­actions (EF_1–3_). The dimeric (EF)_6_ (*i.e.* 2 × EF_1–3_) building blocks pack with the deuterochloro­form guest mol­ecules positioned near each of their corners. The Cl atoms of the latter are disordered over two sets of sites in a 0.53 (2):0.47 (2) ratio.

## Related literature

The solvent-free C_34_H_22_Br_2_N_2_S mol­ecule crystallizes in space group *C*2/*c* exhibiting a layer structure that does not contain (EF)_6_ bricks (Alshahateet *et al.*, 2008 ▶). These bricks are, however, present in five alternative inclusion crystal structures formed by the same host (Alshahateet *et al.*, 2008 ▶). Similar dimeric (EF)_6_ building blocks have also been found in crystal structures of other structurally related racemic diquinoline mol­ecules (Ashmore *et al.*, 2004 ▶, 2009 ▶).
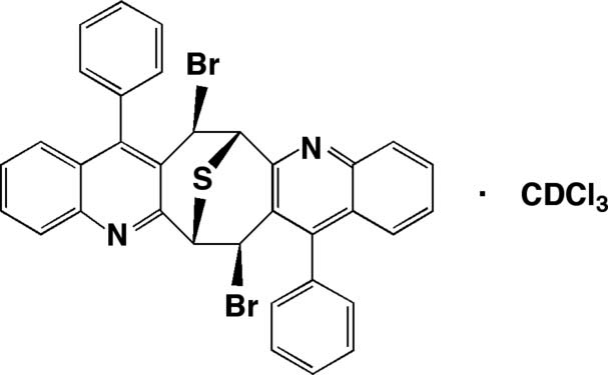


## Experimental

### 

#### Crystal data

C_34_H_22_Br_2_N_2_S·CDCl_3_
*M* *_r_* = 770.81
Triclinic, 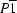

*a* = 10.161 (4) Å
*b* = 10.246 (5) Å
*c* = 15.868 (6) Å
α = 93.88 (3)°
β = 99.43 (3)°
γ = 92.50 (3)°
*V* = 1623.4 (12) Å^3^
*Z* = 2
Mo *K*α radiation
μ = 2.84 mm^−1^
*T* = 294 K
0.26 × 0.24 × 0.12 mm

#### Data collection

Enraf–Nonius CAD-4 diffractometer
Absorption correction: analytical (de Meulenaer & Tompa, 1965 ▶) *T*_min_ = 0.489, *T*_max_ = 0.712
5894 measured reflections
5692 independent reflections
4210 reflections with *I* > 2σ(*I*)
*R*_int_ = 0.032
1 standard reflections frequency: 30 min intensity decay: none

#### Refinement

*R*[*F* ^2^ > 2σ(*F* ^2^)] = 0.045
*wR*(*F* ^2^) = 0.130
*S* = 0.84
5692 reflections
432 parameters
436 restraints
H atoms treated by a mixture of independent and constrained refinement
Δρ_max_ = 0.50 e Å^−3^
Δρ_min_ = −0.64 e Å^−3^

### 

Data collection: *CAD-4 Manual* (Schagen *et al.*, 1989 ▶); cell refinement: *CAD-4 Manual*; data reduction: *CAD-4 Manual*; program(s) used to solve structure: *SIR92* (Altomare *et al.*, 1994 ▶); program(s) used to refine structure: *SHELXL97* (Sheldrick, 2008 ▶); molecular graphics: *ORTEP-3* (Farrugia, 1997 ▶) and *CrystalMaker* (*CrystalMaker*, 2005 ▶); software used to prepare material for publication: *SHELXL97*.

## Supplementary Material

Crystal structure: contains datablocks global, I. DOI: 10.1107/S1600536809032978/hb5037sup1.cif

Structure factors: contains datablocks I. DOI: 10.1107/S1600536809032978/hb5037Isup2.hkl

Additional supplementary materials:  crystallographic information; 3D view; checkCIF report

## Figures and Tables

**Fig. 1.**
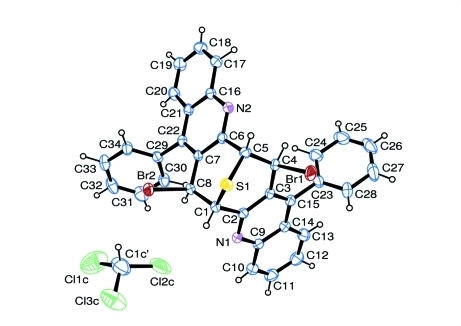
The molecular structure of (I), with ellipsoids drawn at 30% probability level. The second disorder component of the solvent has been omitted for clarity.

**Fig. 2.**
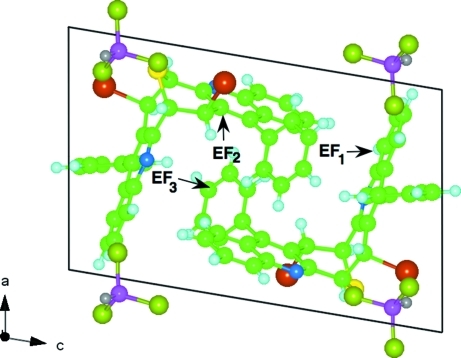
Unit cell diagram of (I) indicating the three different edge-face interactions, EF1–3.

## supplementary crystallographic information

### Comment

The structure of (I).CDCl_3_ is shown in Fig. 1. (I) is chiral, but in the racemic crystal, two molecules related by a centre of inversion form dimeric brick units, associating by means of pairs of three different edge-face interactions (EF_1–3_, Fig. 2). These (EF)_6_ brick units have previously been found to be present in five other crystal structures formed by the same diquinoline host (I) with other solvent inclusion (Alshahateet *et al.*, 2008). In addition, similar dimeric (EF)_6_ building blocks have been found in crystal structures of other inclusion compounds of structurally related racemic diquinoline molecules (Ashmore *et al.*, 2004 and 2009). However, when (I) was previously obtained from CHCl_3_ as solvent-free crystals in space group *C*2/*c* a layer structure resulted that did not contain (EF)_6_ bricks (Alshahateet *et al.*, 2008). Formation of the solvent-free or lattice inclusion crystal forms is probably influenced by the crystallization temperature rather than by the isotopic substitution of the chloroform solvent.

The CDCl_3_ guest is disordered over two sites [occupancies 0.53 (2) and 0.47 (2)] and is located at the corners of the dimeric bricks. Additional lattice stabilization results from host-host and host–guest halogen···halogen and C—H(or D)···halogen interactions.

### Experimental

Racemic 7α,15α-dibromo-8,16-diphenyl-6,7,14,15-tetrahydro-6α,14α-thiacycloocta[1,2-*b*:5,6-*b*']diquinoline was prepared as previously described (Alshahateet *et al.*, 2008) and colourless blocks of (I) were obtained by slow concentration of a deuterochloroform solution.

### Crystal data

**Table:**

C_34_H_22_Br_2_N_2_S·CDCl_3_	*Z* = 2
*M**_r_* = 770.81	*F*(000) = 768
Triclinic, *P*1	*D*_x_ = 1.575 Mg m^−^^3^
Hall symbol: -P 1	Mo *K*α radiation, λ = 0.71073 Å
*a* = 10.161 (4) Å	Cell parameters from 11 reflections
*b* = 10.246 (5) Å	θ = 10.0–11.0°
*c* = 15.868 (6) Å	µ = 2.84 mm^−^^1^
α = 93.88 (3)°	*T* = 294 K
β = 99.43 (3)°	Blocks, colourless
γ = 92.50 (3)°	0.26 × 0.24 × 0.12 mm
*V* = 1623.4 (12) Å^3^	

### Data collection

**Table:**

Enraf–Nonius CAD-4 diffractometer	4210 reflections with *I* > 2σ(*I*)
Radiation source: fine-focus sealed tube	*R*_int_ = 0.032
graphite	θ_max_ = 25.0°, θ_min_ = 2.0°
ω–2θ scans	*h* = −12→11
Absorption correction: analytical (de Meulenaer & Tompa, 1965)	*k* = −12→12
*T*_min_ = 0.489, *T*_max_ = 0.712	*l* = 0→18
5894 measured reflections	1 standard reflections every 30 min
5692 independent reflections	intensity decay: none

### Refinement

**Table:**

Refinement on *F*^2^	Primary atom site location: structure-invariant direct methods
Least-squares matrix: full	Secondary atom site location: difference Fourier map
*R*[*F*^2^ > 2σ(*F*^2^)] = 0.045	Hydrogen site location: inferred from neighbouring sites
*wR*(*F*^2^) = 0.130	H atoms treated by a mixture of independent and constrained refinement
*S* = 0.84	*w* = 1/[σ^2^(*F*_o_^2^) + (0.1*P*)^2^ + *P*] where *P* = (*F*_o_^2^ + 2*F*_c_^2^)/3
5692 reflections	(Δ/σ)_max_ < 0.001
432 parameters	Δρ_max_ = 0.50 e Å^−^^3^
436 restraints	Δρ_min_ = −0.64 e Å^−^^3^

### Special details

**Table:**

Geometry. All e.s.d.'s (except the e.s.d. in the dihedral angle between two l.s. planes) are estimated using the full covariance matrix. The cell e.s.d.'s are taken into account individually in the estimation of e.s.d.'s in distances, angles and torsion angles; correlations between e.s.d.'s in cell parameters are only used when they are defined by crystal symmetry. An approximate (isotropic) treatment of cell e.s.d.'s is used for estimating e.s.d.'s involving l.s. planes.
Refinement. Refinement of *F*^2^ against ALL reflections. The weighted *R*-factor *wR* and goodness of fit *S* are based on *F*^2^, conventional *R*-factors *R* are based on *F*, with *F* set to zero for negative *F*^2^. The threshold expression of *F*^2^ > σ(*F*^2^) is used only for calculating *R*-factors(gt) *etc*. and is not relevant to the choice of reflections for refinement. *R*-factors based on *F*^2^ are statistically about twice as large as those based on *F*, and *R*- factors based on ALL data will be even larger. The orientational disorder of CDCl_3_ (about the C1C'-H1C' bond) over two major sites (in 52:48 ratio) was modelled using PART instruction in *SHELXL97*. The molecular geometry of CDCl_3_ was restrained to have values within the observed range; anisotropic thermal parameters of the disordered atoms were also restrained using the same program.

### Fractional atomic coordinates and isotropic or equivalent isotropic displacement parameters (Å^2^)

**Table:**

	*x*	*y*	*z*	*U*_iso_*/*U*_eq_	Occ. (<1)
C1C'	0.0673 (7)	0.8408 (6)	0.8664 (4)	0.114 (2)	
D1C'	0.0960	0.7680	0.9030	0.137*	
Cl1C	0.1613 (13)	0.9741 (11)	0.9107 (9)	0.197 (5)	0.53 (2)
Cl2C	0.0864 (11)	0.7870 (9)	0.7614 (4)	0.163 (4)	0.53 (2)
Cl3C	−0.1006 (7)	0.8557 (12)	0.8705 (10)	0.142 (3)	0.53 (2)
Cl1'	0.1385 (9)	0.9940 (6)	0.9039 (8)	0.141 (3)	0.47 (2)
Cl2'	0.0872 (12)	0.835 (2)	0.7613 (5)	0.208 (5)	0.47 (2)
Cl3'	−0.0929 (12)	0.8162 (17)	0.8811 (15)	0.198 (6)	0.47 (2)
Br1	0.12588 (5)	0.05682 (4)	0.58378 (3)	0.05792 (17)	
Br2	0.22328 (4)	0.53263 (4)	0.90496 (3)	0.04953 (15)	
N1	0.1465 (3)	0.5410 (3)	0.6081 (2)	0.0426 (8)	
N2	0.4967 (3)	0.2104 (3)	0.7913 (2)	0.0383 (7)	
S1	0.11551 (10)	0.26209 (10)	0.76288 (7)	0.0448 (3)	
C1	0.1492 (4)	0.4236 (4)	0.7312 (2)	0.0390 (9)	
C2	0.1736 (4)	0.4270 (4)	0.6392 (2)	0.0369 (8)	
C3	0.2294 (4)	0.3238 (4)	0.5954 (2)	0.0368 (8)	
C4	0.2600 (4)	0.1981 (4)	0.6354 (2)	0.0386 (8)	
C5	0.2671 (4)	0.2031 (4)	0.7328 (3)	0.0391 (8)	
C6	0.3919 (4)	0.2822 (4)	0.7772 (2)	0.0349 (8)	
C7	0.3956 (4)	0.4196 (3)	0.7990 (2)	0.0329 (8)	
C8	0.2695 (4)	0.4926 (4)	0.7892 (2)	0.0383 (9)	
C9	0.1710 (4)	0.5593 (4)	0.5278 (3)	0.0430 (9)	
C10	0.1405 (5)	0.6812 (4)	0.4937 (3)	0.0531 (11)	
H10	0.1031	0.7466	0.5264	0.064*	
C11	0.1644 (5)	0.7041 (5)	0.4151 (3)	0.0625 (12)	
H11	0.1433	0.7858	0.3929	0.075*	
C12	0.2197 (5)	0.6107 (5)	0.3651 (3)	0.0650 (13)	
H12	0.2363	0.6294	0.3099	0.078*	
C13	0.2499 (5)	0.4924 (5)	0.3959 (3)	0.0571 (11)	
H13	0.2862	0.4286	0.3614	0.069*	
C14	0.2278 (4)	0.4640 (4)	0.4783 (3)	0.0447 (9)	
C15	0.2573 (4)	0.3430 (4)	0.5148 (3)	0.0421 (9)	
C16	0.6163 (4)	0.2717 (4)	0.8271 (2)	0.0382 (8)	
C17	0.7270 (4)	0.1935 (4)	0.8455 (3)	0.0471 (10)	
H17	0.7161	0.1011	0.8340	0.056*	
C18	0.8490 (4)	0.2505 (5)	0.8796 (3)	0.0555 (11)	
H18	0.9224	0.1969	0.8931	0.067*	
C19	0.8689 (4)	0.3867 (5)	0.8953 (3)	0.0579 (12)	
H19	0.9556	0.4253	0.9170	0.070*	
C20	0.7626 (4)	0.4635 (4)	0.8792 (3)	0.0492 (10)	
H20	0.7764	0.5557	0.8905	0.059*	
C21	0.6328 (4)	0.4095 (4)	0.8463 (2)	0.0382 (8)	
C22	0.5179 (4)	0.4835 (4)	0.8319 (2)	0.0373 (8)	
C23	0.3176 (5)	0.2394 (4)	0.4643 (3)	0.0479 (10)	
C24	0.4528 (5)	0.2252 (5)	0.4776 (3)	0.0635 (13)	
H24	0.5103	0.2831	0.5181	0.076*	
C25	0.5059 (7)	0.1250 (7)	0.4310 (4)	0.0881 (19)	
H25	0.5993	0.1139	0.4411	0.106*	
C26	0.4250 (8)	0.0445 (6)	0.3721 (4)	0.0868 (18)	
H26	0.4616	−0.0228	0.3405	0.104*	
C27	0.2903 (8)	0.0594 (6)	0.3575 (4)	0.0859 (17)	
H27	0.2339	0.0020	0.3159	0.103*	
C28	0.2352 (6)	0.1577 (5)	0.4029 (3)	0.0686 (14)	
H28	0.1417	0.1687	0.3919	0.082*	
C29	0.5295 (4)	0.6274 (4)	0.8582 (3)	0.0404 (9)	
C30	0.5095 (5)	0.7195 (4)	0.7992 (3)	0.0535 (11)	
H30	0.4902	0.6929	0.7398	0.064*	
C31	0.5173 (6)	0.8518 (5)	0.8265 (4)	0.0708 (14)	
H31	0.5026	0.9153	0.7855	0.085*	
C32	0.5463 (6)	0.8915 (5)	0.9125 (4)	0.0700 (14)	
H32	0.5513	0.9821	0.9306	0.084*	
C33	0.5677 (5)	0.8008 (5)	0.9714 (3)	0.0631 (13)	
H33	0.5890	0.8278	1.0308	0.076*	
C34	0.5582 (5)	0.6691 (4)	0.9444 (3)	0.0498 (10)	
H34	0.5716	0.6060	0.9858	0.060*	
H1	0.080 (4)	0.472 (4)	0.739 (2)	0.038 (11)*	
H4	0.349 (4)	0.158 (4)	0.622 (2)	0.039 (10)*	
H5	0.273 (4)	0.120 (4)	0.751 (3)	0.038 (10)*	
H8	0.284 (4)	0.577 (4)	0.775 (2)	0.036 (10)*	

### Atomic displacement parameters (Å^2^)

**Table:**

	*U*^11^	*U*^22^	*U*^33^	*U*^12^	*U*^13^	*U*^23^
C1C'	0.137 (5)	0.087 (4)	0.128 (5)	0.040 (4)	0.037 (5)	0.015 (4)
Cl1C	0.137 (6)	0.205 (9)	0.220 (9)	−0.029 (6)	−0.043 (7)	0.019 (6)
Cl2C	0.225 (8)	0.167 (5)	0.118 (4)	0.147 (6)	0.045 (4)	0.039 (3)
Cl3C	0.106 (4)	0.095 (6)	0.236 (8)	0.010 (3)	0.065 (4)	0.009 (5)
Cl1'	0.118 (5)	0.063 (3)	0.256 (10)	0.012 (3)	0.068 (6)	0.006 (4)
Cl2'	0.159 (8)	0.369 (16)	0.091 (4)	0.013 (9)	−0.004 (4)	0.044 (6)
Cl3'	0.179 (8)	0.110 (9)	0.323 (14)	−0.048 (6)	0.106 (9)	0.018 (8)
Br1	0.0681 (3)	0.0409 (3)	0.0561 (3)	−0.0073 (2)	−0.0066 (2)	−0.0104 (2)
Br2	0.0486 (3)	0.0618 (3)	0.0377 (2)	0.0112 (2)	0.00862 (18)	−0.00863 (19)
N1	0.0443 (19)	0.0419 (19)	0.0382 (18)	0.0076 (15)	−0.0018 (15)	−0.0026 (14)
N2	0.0404 (17)	0.0361 (17)	0.0378 (17)	0.0034 (14)	0.0062 (14)	−0.0016 (14)
S1	0.0413 (6)	0.0459 (6)	0.0466 (6)	−0.0004 (4)	0.0075 (4)	0.0001 (5)
C1	0.039 (2)	0.041 (2)	0.036 (2)	0.0077 (17)	0.0047 (17)	−0.0043 (16)
C2	0.0314 (19)	0.039 (2)	0.038 (2)	0.0019 (15)	0.0013 (15)	−0.0015 (16)
C3	0.035 (2)	0.036 (2)	0.0352 (19)	−0.0008 (15)	−0.0010 (16)	−0.0053 (15)
C4	0.042 (2)	0.034 (2)	0.036 (2)	−0.0020 (16)	−0.0009 (16)	−0.0065 (16)
C5	0.040 (2)	0.032 (2)	0.044 (2)	0.0012 (16)	0.0022 (17)	0.0042 (17)
C6	0.039 (2)	0.037 (2)	0.0287 (18)	0.0028 (15)	0.0078 (15)	0.0004 (15)
C7	0.0392 (19)	0.0315 (18)	0.0279 (18)	0.0037 (15)	0.0062 (15)	−0.0027 (14)
C8	0.041 (2)	0.041 (2)	0.0328 (19)	0.0089 (17)	0.0073 (16)	−0.0042 (17)
C9	0.046 (2)	0.038 (2)	0.041 (2)	0.0068 (17)	−0.0061 (17)	−0.0001 (16)
C10	0.062 (3)	0.045 (2)	0.051 (3)	0.008 (2)	0.002 (2)	0.0029 (19)
C11	0.072 (3)	0.054 (3)	0.059 (3)	0.005 (2)	−0.003 (2)	0.016 (2)
C12	0.075 (3)	0.073 (3)	0.047 (3)	0.002 (3)	0.008 (2)	0.017 (2)
C13	0.067 (3)	0.060 (3)	0.046 (2)	0.012 (2)	0.013 (2)	0.006 (2)
C14	0.046 (2)	0.049 (2)	0.038 (2)	0.0064 (18)	0.0017 (18)	0.0002 (17)
C15	0.039 (2)	0.046 (2)	0.038 (2)	0.0046 (17)	0.0002 (17)	−0.0051 (16)
C16	0.040 (2)	0.042 (2)	0.033 (2)	0.0051 (16)	0.0083 (16)	0.0000 (16)
C17	0.046 (2)	0.049 (2)	0.048 (2)	0.0093 (18)	0.0102 (19)	−0.0002 (19)
C18	0.036 (2)	0.072 (3)	0.059 (3)	0.013 (2)	0.009 (2)	0.000 (2)
C19	0.035 (2)	0.075 (3)	0.062 (3)	0.001 (2)	0.010 (2)	−0.009 (2)
C20	0.042 (2)	0.052 (2)	0.053 (3)	−0.0040 (18)	0.0112 (19)	−0.007 (2)
C21	0.039 (2)	0.045 (2)	0.032 (2)	0.0013 (16)	0.0108 (16)	−0.0037 (16)
C22	0.045 (2)	0.039 (2)	0.0282 (18)	0.0000 (16)	0.0089 (16)	−0.0004 (15)
C23	0.061 (3)	0.044 (2)	0.040 (2)	0.0096 (19)	0.0114 (19)	−0.0020 (18)
C24	0.067 (3)	0.070 (3)	0.057 (3)	0.021 (2)	0.015 (2)	0.005 (2)
C25	0.096 (4)	0.112 (5)	0.066 (4)	0.060 (4)	0.027 (3)	0.008 (3)
C26	0.148 (5)	0.069 (4)	0.057 (3)	0.051 (4)	0.042 (4)	0.008 (3)
C27	0.137 (5)	0.063 (3)	0.058 (3)	0.009 (4)	0.025 (3)	−0.018 (3)
C28	0.086 (4)	0.068 (3)	0.048 (3)	0.009 (3)	0.008 (2)	−0.020 (2)
C29	0.038 (2)	0.038 (2)	0.045 (2)	0.0015 (16)	0.0084 (17)	−0.0039 (17)
C30	0.070 (3)	0.045 (2)	0.046 (2)	−0.004 (2)	0.015 (2)	0.0007 (19)
C31	0.099 (4)	0.043 (3)	0.077 (3)	0.004 (3)	0.029 (3)	0.016 (2)
C32	0.096 (4)	0.038 (3)	0.081 (3)	−0.004 (2)	0.036 (3)	−0.010 (2)
C33	0.078 (3)	0.051 (3)	0.057 (3)	−0.008 (2)	0.015 (3)	−0.018 (2)
C34	0.062 (3)	0.043 (2)	0.042 (2)	0.000 (2)	0.008 (2)	−0.0035 (18)

### Geometric parameters (Å, °)

**Table:**

C1C'—Cl1C	1.676 (8)	C13—H13	0.9500
C1C'—Cl2C	1.765 (8)	C14—C15	1.427 (6)
C1C'—Cl3C	1.730 (9)	C15—C23	1.500 (6)
C1C'—Cl1'	1.724 (9)	C16—C17	1.412 (6)
C1C'—Cl2'	1.711 (10)	C16—C21	1.421 (6)
C1C'—Cl3'	1.692 (12)	C17—C18	1.358 (6)
C1C'—D1C'	1.0000	C17—H17	0.9500
Br1—C4	1.981 (4)	C18—C19	1.400 (7)
Br2—C8	1.990 (4)	C18—H18	0.9500
N1—C2	1.322 (5)	C19—C20	1.363 (6)
N1—C9	1.361 (5)	C19—H19	0.9500
N2—C6	1.319 (5)	C20—C21	1.411 (6)
N2—C16	1.361 (5)	C20—H20	0.9500
S1—C1	1.796 (4)	C21—C22	1.415 (5)
S1—C5	1.802 (4)	C22—C29	1.499 (5)
C1—C8	1.519 (6)	C23—C24	1.370 (7)
C1—C2	1.523 (5)	C23—C28	1.382 (7)
C1—H1	0.89 (4)	C24—C25	1.402 (7)
C2—C3	1.416 (5)	C24—H24	0.9500
C3—C15	1.378 (6)	C25—C26	1.348 (9)
C3—C4	1.497 (5)	C25—H25	0.9500
C4—C5	1.532 (6)	C26—C27	1.367 (9)
C4—H4	1.06 (4)	C26—H26	0.9500
C5—C6	1.517 (5)	C27—C28	1.390 (7)
C5—H5	0.92 (4)	C27—H27	0.9500
C6—C7	1.425 (5)	C28—H28	0.9500
C7—C22	1.385 (5)	C29—C30	1.372 (6)
C7—C8	1.503 (5)	C29—C34	1.386 (6)
C8—H8	0.92 (4)	C30—C31	1.389 (6)
C9—C14	1.413 (6)	C30—H30	0.9500
C9—C10	1.422 (6)	C31—C32	1.377 (7)
C10—C11	1.343 (7)	C31—H31	0.9500
C10—H10	0.9500	C32—C33	1.362 (7)
C11—C12	1.396 (7)	C32—H32	0.9500
C11—H11	0.9500	C33—C34	1.382 (6)
C12—C13	1.365 (7)	C33—H33	0.9500
C12—H12	0.9500	C34—H34	0.9500
C13—C14	1.410 (6)		
			
Cl1C—C1C'—Cl2C	116.3 (8)	C14—C13—H13	119.6
Cl1C—C1C'—Cl3C	112.5 (7)	C13—C14—C9	118.7 (4)
Cl3C—C1C'—Cl2C	109.4 (7)	C13—C14—C15	123.9 (4)
Cl2'—C1C'—Cl1'	101.7 (9)	C9—C14—C15	117.4 (4)
Cl3'—C1C'—Cl1'	114.3 (7)	C3—C15—C14	119.4 (4)
Cl3'—C1C'—Cl2'	114.2 (10)	C3—C15—C23	121.6 (4)
Cl1C—C1C'—D1C'	106.0	C14—C15—C23	119.0 (4)
Cl2C—C1C'—D1C'	106.0	N2—C16—C17	117.8 (4)
Cl3C—C1C'—D1C'	106.0	N2—C16—C21	122.5 (3)
Cl1'—C1C'—D1C'	115.4	C17—C16—C21	119.7 (4)
Cl2'—C1C'—D1C'	120.7	C18—C17—C16	119.9 (4)
Cl3'—C1C'—D1C'	91.2	C18—C17—H17	120.0
C2—N1—C9	117.7 (3)	C16—C17—H17	120.0
C6—N2—C16	118.1 (3)	C17—C18—C19	121.4 (4)
C1—S1—C5	92.66 (19)	C17—C18—H18	119.3
C8—C1—C2	107.7 (3)	C19—C18—H18	119.3
C8—C1—S1	111.4 (3)	C20—C19—C18	119.4 (4)
C2—C1—S1	114.3 (3)	C20—C19—H19	120.3
C8—C1—H1	105 (3)	C18—C19—H19	120.3
C2—C1—H1	109 (3)	C19—C20—C21	121.8 (4)
S1—C1—H1	109 (3)	C19—C20—H20	119.1
N1—C2—C3	124.4 (4)	C21—C20—H20	119.1
N1—C2—C1	111.9 (3)	C20—C21—C22	124.3 (4)
C3—C2—C1	123.5 (3)	C20—C21—C16	117.7 (4)
C15—C3—C2	118.1 (4)	C22—C21—C16	117.9 (3)
C15—C3—C4	120.6 (3)	C7—C22—C21	119.1 (3)
C2—C3—C4	121.4 (3)	C7—C22—C29	121.4 (3)
C3—C4—C5	115.5 (3)	C21—C22—C29	119.4 (3)
C3—C4—Br1	110.4 (3)	C24—C23—C28	119.7 (4)
C5—C4—Br1	107.7 (3)	C24—C23—C15	121.1 (4)
C3—C4—H4	114 (2)	C28—C23—C15	119.2 (4)
C5—C4—H4	107 (2)	C23—C24—C25	119.7 (5)
Br1—C4—H4	101 (2)	C23—C24—H24	120.1
C6—C5—C4	110.1 (3)	C25—C24—H24	120.1
C6—C5—S1	113.3 (3)	C26—C25—C24	120.3 (6)
C4—C5—S1	110.8 (3)	C26—C25—H25	119.8
C6—C5—H5	107 (2)	C24—C25—H25	119.8
C4—C5—H5	110 (2)	C25—C26—C27	120.2 (5)
S1—C5—H5	106 (2)	C25—C26—H26	119.9
N2—C6—C7	123.9 (3)	C27—C26—H26	119.9
N2—C6—C5	112.6 (3)	C26—C27—C28	120.6 (6)
C7—C6—C5	123.4 (3)	C26—C27—H27	119.7
C22—C7—C6	118.2 (3)	C28—C27—H27	119.7
C22—C7—C8	120.9 (3)	C23—C28—C27	119.3 (6)
C6—C7—C8	120.9 (3)	C23—C28—H28	120.3
C7—C8—C1	115.8 (3)	C27—C28—H28	120.3
C7—C8—Br2	108.5 (3)	C30—C29—C34	118.7 (4)
C1—C8—Br2	109.1 (3)	C30—C29—C22	121.9 (4)
C7—C8—H8	112 (2)	C34—C29—C22	119.4 (4)
C1—C8—H8	112 (2)	C29—C30—C31	120.0 (4)
Br2—C8—H8	99 (2)	C29—C30—H30	120.0
N1—C9—C14	123.0 (4)	C31—C30—H30	120.0
N1—C9—C10	118.0 (4)	C32—C31—C30	120.5 (5)
C14—C9—C10	119.0 (4)	C32—C31—H31	119.7
C11—C10—C9	120.0 (4)	C30—C31—H31	119.7
C11—C10—H10	120.0	C33—C32—C31	120.0 (4)
C9—C10—H10	120.0	C33—C32—H32	120.0
C10—C11—C12	121.7 (5)	C31—C32—H32	120.0
C10—C11—H11	119.2	C32—C33—C34	119.6 (5)
C12—C11—H11	119.2	C32—C33—H33	120.2
C13—C12—C11	119.8 (5)	C34—C33—H33	120.2
C13—C12—H12	120.1	C33—C34—C29	121.3 (4)
C11—C12—H12	120.1	C33—C34—H34	119.4
C12—C13—C14	120.7 (5)	C29—C34—H34	119.4
C12—C13—H13	119.6		
